# Assistant diagnosis with Chinese electronic medical records based on CNN and BiLSTM with phrase-level and word-level attentions

**DOI:** 10.1186/s12859-020-03554-x

**Published:** 2020-06-05

**Authors:** Tong Wang, Ping Xuan, Zonglin Liu, Tiangang Zhang

**Affiliations:** 1 School of Computer Science and Technology, Heilongjiang University, Harbin, 150080 China; 2 School of Mathematical Science, Heilongjiang University, Harbin, 150080 China

**Keywords:** EMR-related disease prediction, Convolutional neural network, Bidirectional long short-term memory, Attention at phrase level, Attention at word level

## Abstract

**Background:**

Inferring diseases related to the patient’s electronic medical records (EMRs) is of great significance for assisting doctor diagnosis. Several recent prediction methods have shown that deep learning-based methods can learn the deep and complex information contained in EMRs. However, they do not consider the discriminative contributions of different phrases and words. Moreover, local information and context information of EMRs should be deeply integrated.

**Results:**

A new method based on the **f**usion of a convolutional neural network (**CN**N) and **b**idirectional **l**ong short-term memory (**B**i**L**STM) with **a**ttention mechanisms is proposed for predicting a disease related to a given EMR, and it is referred to as **FCNBLA**. FCNBLA deeply integrates local information, context information of the word sequence and more informative phrases and words. A novel framework based on deep learning is developed to learn the local representation, the context representation and the combination representation. The left side of the framework is constructed based on CNN to learn the local representation of adjacent words. The right side of the framework based on BiLSTM focuses on learning the context representation of the word sequence. Not all phrases and words contribute equally to the representation of an EMR meaning. Therefore, we establish the attention mechanisms at the phrase level and word level, and the middle module of the framework learns the combination representation of the enhanced phrases and words. The macro average f-score and accuracy of FCNBLA achieved 91.29 and 92.78%, respectively.

**Conclusion:**

The experimental results indicate that FCNBLA yields superior performance compared with several state-of-the-art methods. The attention mechanisms and combination representations are also confirmed to be helpful for improving FCNBLA’s prediction performance. Our method is helpful for assisting doctors in diagnosing diseases in patients.

## Background

Electronic medical records (EMRs), which record patient phenotypes and treatments, are an underutilized data source. Extracting useful information and predicting diseases using EMRs to assist doctors in disease determination and timely treatment of patients is one of the goals of intelligent medical construction [1–3], which can not only help us better understand the clinical manifestations of various diseases [4–6] but also reduce medical errors to improve the health of patients and improve the work efficiency of doctors [7–9].

The previous methods for predicting diseases related to information in EMRs can be roughly grouped into three categories. The methods in the first category are rule-based, which can also be called expert systems. Expert systems are designed to address problems by utilizing the knowledge and experience of human experts [10]. They perform rule matching on each input EMR to select the disease that best fits these rules to implement the corresponding diagnosis for patients. These methods have achieved great success in the field of medical aided diagnosis [11–13]. However, as time goes on, there have been increasingly more cases, and the data are no longer relatively structured and constrained but tend to be multistructured and unstructured. Therefore, rulemaking has become infeasible.

Methods in the second category construct shallow models based on machine learning. Such methods have achieved considerable success in the fields of text classification [14–16], legal prediction [17–19] and intelligent medical systems [20–22]. For example, some common techniques are utilized on public medical datasets for predicting diseases, such as support vector machines [23–25], random forests [26, 27], and logistic regression [28], and they achieve good predictive results. However, these methods have certain limitations in feature extraction. They usually need to artificially design certain features as input for machine learning and cannot capture the deep and complex internal information of data.

**Fig. 1:**
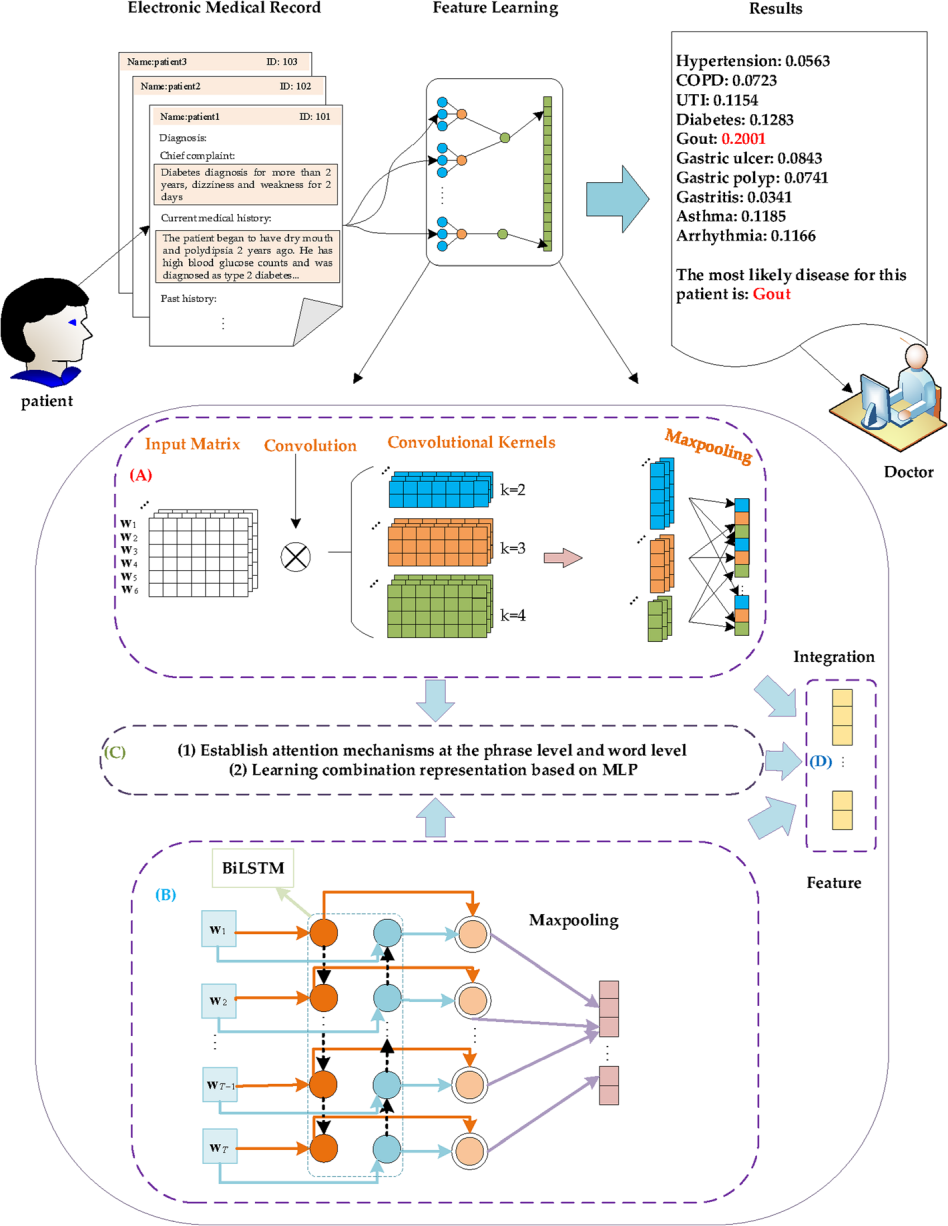
Schematic diagram for predicting diseases related to EMRS. **a** Extracted local information from several adjacent words. **b** Extracted context information for words in EMRs. **c** Establish attention mechanisms at the word level and phrase level and deeply integrate local and context information to obtain combination information. **d** Fully learn local information, context information and combination information

The methods in the third category are based on deep learning. In recent years, deep learning has achieved the most advanced effects on various natural language processing tasks, such as machine translation [29, 30], sentiment analysis [31, 32], speech recognition [33, 34] and language modeling [35–37]. Moreover, in the medical field, experiments have proven that deep learning methods outperform state-of-the-art traditional predictive models in all cases with electronic health record (EHR) data. For example, Cheng et al. [38] proposed a prediction method based on convolutional neural network (CNN) for the risk prediction of EHR. Nguyen et al. [39] introduced a CNN model for predicting the probability of readmission. Choi et al. [40] introduced a shallow recurrent neural network (RNN) model to predict diagnoses and medications. Li et al. [41] provided a transformer-based model to predict diseases in the future. Due to the advantages of deep learning in cases of using EHR data, some deep learning-based models were applied to the diagnosis of Chinese electronic medical records. CNN have strong capabilities in feature extraction and expression [42, 43]. For example, methods based on CNN were proposed by Yang et al. and Chen et al. to predict diseases [10, 44]. They focused on extracting local information from adjacent words. However, these methods failed to consider the context information of the word sequence. Usama et al. and Hao et al. proposed prediction methods based on a recurrent convolutional neural network (RCNN) [45, 46], which learns the local information of the word context. However, they did not fully exploit the whole context and local information. In addition, the previous methods do not discriminate the different contributions of different phrases and words. In our study, a novel method based on CNN and bidirectional long short-term memory (BiLSTM) with attention mechanisms is proposed for obtaining the latent representations of EMRs, which we refer to as FCNBLA (Fig. 1). FCNBLA fully integrates local information formed by several adjacent words, context information of the whole sentence, and enhanced phrase and word information. Figure 1a is dedicated to feature extraction from adjacent words of an EMR to obtain their local representation. In Fig. 1b, the context representation is learned from the whole EMR based on BiLSTM. In Fig. 1c, each phrase and word are assigned different weights by applying attention mechanisms, which may discriminate their different contributions for predicting diseases related to EMRs. The experimental results indicate that FCNBLA outperforms several state-of-the-art methods for predicting diseases.

## Methods

### Datasets for disease prediction related to EMRs

The EMR dataset we use comes from previous work on disease prediction [10]. The original 18,625 EMRs were originally collected from Huangshi Central Hospital in China. The dataset contains the 10 most common diseases: *diabetes, hypertension, chronic obstructive pulmonary disease (COPD), arrhythmia, asthma, gastritis, gastric polyps, gout, gastric ulcers and urinary tract infection (UTI)*. Each EMR contains 18 items: initial diagnosis on admission, chief complaint, history of surgery, vital signs, specialist condition, general condition, allergic history, nutritional status, suicidal tendency, specialist examination, history of surgical trauma, complications, current medical history, fertility history, auxiliary examination, personal history, past medical history, and family history. Among them, initial diagnosis on admission is a disease related to an EMR, and the remaining 17 items record the patient’s condition. However, 24 EMRs only included the initial diagnosis on admission but did not include any of the remaining 17 items, so we removed them. We used the remaining 18,601 EMRs as our experimental data.

We selected 70% of the 18,601 EMRs as the training set to train the model, selected 10% as the validation set to adjust the model parameters, and selected 20% to test performance of the model. The distributions of the training, validation and testing sets are consistent with the original data distributions of 10 diseases. For the 10 diseases, their training, validation, and testing data distributions are shown in Table 1.

**Table 1:** Number of EMRs related to each disease in the training, validation and test sets

Diseases	Training set	Validation set	Test set
diabetes	3952	564	1129
hypertension	2763	395	789
COPD	2310	330	660
arrhythmia	1016	145	290
asthma	753	108	215
gastritis	748	107	214
gastric polyps	511	73	146
gout	460	66	131
gastric ulcers	305	44	87
UTI	203	29	58

### Disease prediction model

**Fig. 2:**
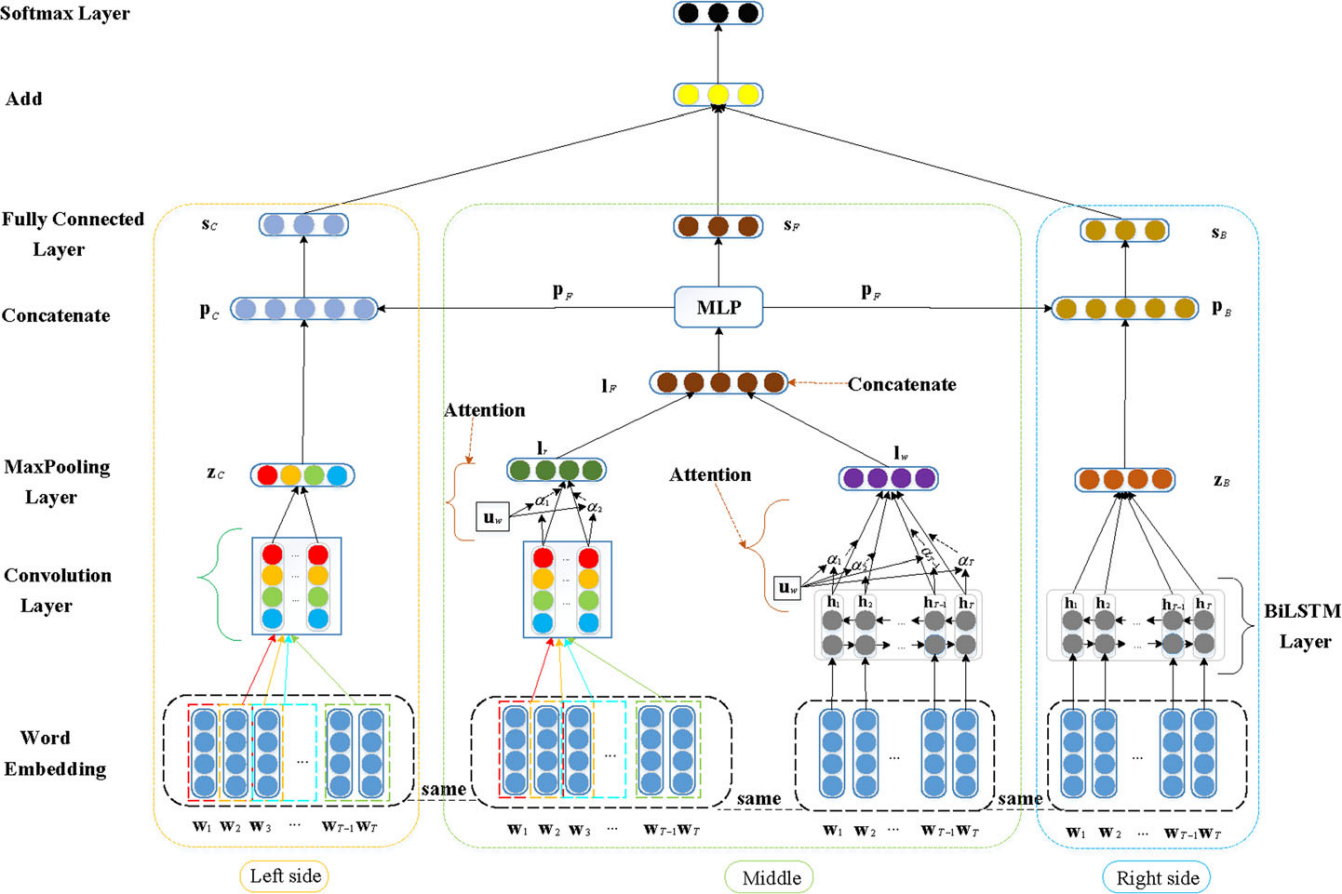
The overall framework of the model for learning the potential representation of EMRs. The left of the framework is the CNN module, the right is the BiLSTM module, and the middle is the attention module

In this section, we describe our prediction model for learning the latent representations of EMRs and predicting diseases related to EMRs. Figure 2 shows the overall architecture of the model, which involves three major network modules. The left side is the convolutional module, which learns the local representation of a given EMR. The BiLSTM module on the right side learns the context representation of the EMR. The middle part is the fusion of local information and context information of the EMR, and its fusion representation is obtained. For disease prediction, we design a combination strategy to estimate the final association score between a disease and the EMR.

#### Word embedding layer

We use word embeddings as a representation of each EMR in the input layer. The word embedding layer can be simply understood as a look-up operation; that is, it reads a one-hot vector, **e**_*t*_  ∈ *R*^∣*V*∣^, for a word and maps it to a dense vector of *d* dimensions, **x**_*t*_ = (*x*_1_, *x*_2_, …, *x*_*d*_) as an input of the disease prediction model. The weight matrix of the word embeddings is **H**  ∈ *R*^*d* ×  ∣ *V*∣^, which is randomly initialized. We fine-tune the initial word embeddings, modifying them during gradient updates of the neural network model by backpropagating gradients. We have the following formula:

1 $$ {\mathbf{x}}_t={\mathbf{He}}_t, $$

where *V* denotes a series of words and |*V*| is the size of the vocabulary.

#### Convolutional module on the left

The CNN proposed by Lecun et al. [47] can automatically learn feature representations. The CNN architecture is composed of three different layers: the convolutional layer, the pooling layer and the fully connected layer, as shown on the left side of Fig. 2.

An EMR consisting of *T* words to be classified is fed into the word embedding layer, *T* words are converted into vectors, and then an embedding matrix **I**  ∈ *R*^*T* × *d*^ is formed as the input of the CNN. The convolutional layer and pooling layer are the core of the CNN. The CNN used in our framework consists of a convolutional layer followed by a max pooling layer. For the convolutional layer, we use 3 filters with different heights to slide across **I** and there are 50 filters for each height. Assume the height of a filter is *k*, which means the filter operates on the adjacent *k* words and the width of each filter is the same as the dimension of each input word embedding matrix and the outputs of the convolutional layer are feature maps.

The pooling layer may reduce the parameters of the neural network while maintaining the attributes of the word sequence so that the model can be effectively prevented from overfitting [10]. The pooling operation focuses on computing the max or average of the local regions. In this paper, we use the max pooling operation for each feature **Z**. After the pooling operation calculation is completed, all the extracted features are concatenated to form a local representation **z**_*C*_ of an EMR.

#### BiLSTM module on the right

LSTM was proposed by Hochreiter et al. [48] to solve the gradient vanishing/exploding problem of RNN. However, LSTM can only obtain information from past words. For the task of determining the disease that an EMR is related to, it is very useful to obtain the past and future context information because each word of an EMR is semantically related to other words. The BiLSTM proposed by Dyer et al. [49] extended the unidirectional LSTM by introducing a second hidden layer, and the connections between hidden layers flow in reverse chronological order. Therefore, BiLSTM can be used to capture context information of an EMR. As shown in Fig. 2, on the right side of the framework, the BiLSTM contains two subnetworks: the forward LSTM is used for obtaining the forward sequence context ${\overrightarrow{\mathbf{h}}}_t$, and the backward LSTM obtains the backward sequence context ${\overleftarrow{\mathbf{h}}}_t$. The final hidden state **h**_*t*_ of each word is the concatenation of ${\overrightarrow{\mathbf{h}}}_t$ and ${\overleftarrow{\mathbf{h}}}_t$.

#### Attention module on the middle

In our model, the attention module is used to learn which words or phrases are more important for the representation of an EMR. Therefore, the module consists of the attention mechanism at the phrase level and the one at the word level.

##### Attention at the phrase level

**Z** obtained by the left convolutional module is composed of *N* (1 ≤ *N* ≤ *T* − *k* + 1) rows. We call each row of **Z** a phrase vector, which contains the convolution results from the *j* filters performing convolution operations on a sequence of *k* word embeddings. **Z**_*i*_ is the *i*-th row of **Z**. Different phrases usually have different contributions to the representation of the EMR. Thus, we establish the attention mechanism for each phrase vector **Z**_*i*_ to generate the final attention representation. **Z**_*i*_ is assigned an attention weight *β*_*i*_, and *β*_*i*_ is defined as follows:

2 $$ {\mathbf{v}}_i=\tanh \left({\mathbf{W}}_r{\mathbf{Z}}_i+{\mathbf{b}}_r\right), $$

3 $$ {\beta}_i=\frac{\exp \left({\mathbf{v}}_i^{\top }{\mathbf{u}}_p\right)}{\sum \limits_{l=1}^N\exp \left({\mathbf{v}}_l^{\top }{\mathbf{u}}_p\right)}, $$

where **W**_*r*_ is a weight matrix, **b**_*r*_ is a bias vector, and **u**_*p*_ is a phrase-level context vector. **v**_*i*_ is the feature representation of **Z**_*i*_, which is obtained by feeding **Z**_*i*_ into a one-layer multilayer perceptron (MLP). *β*_*i*_ is a standardized importance weight of **Z**_*i*_ and *N* is the number of rows of the feature map **Z** obtained by the convolutional layer. The phrase context vector **u**_*p*_ is randomly initialized and updated during the training process. We aggregate the representations of those informative phrases to form the enhanced local phrase information of an EMR, which is represented as follows:

4 $$ {\mathbf{l}}_r=\sum \limits_{i=1}^N{\beta}_i\kern0.5em {Z}_i. $$

##### Attention at the word level

Different words also contribute differently to the representation of an EMR. Therefore, we establish a word level on the hidden state **h**_*t*_ (1 ≤ *t* ≤ *T*) to generate the final attention representation. The attention weight at the word level is given as follows:

5 $$ {\mathbf{u}}_t=\tanh \left({\mathbf{W}}_c{\mathbf{h}}_t+{\mathbf{b}}_c\right), $$

6 $$ {\alpha}_t=\frac{\exp \left({\mathbf{u}}_t^{\top }{\mathbf{u}}_w\right)}{\sum \limits_{j=1}^T\exp \left({\mathbf{u}}_j^{\top }{\mathbf{u}}_w\right)}, $$

where **W**_*c*_ is a weight matrix, **b**_*c*_ indicates a bias vector and **u**_*w*_ is a word-level context vector. **u**_*t*_ is a hidden representation of **h**_*t*_ and *α*_*t*_ is a normalized attention weight of **h**_*t*_. The important context information of the whole sentence is represented as **l**_*w*_,

7 $$ {\mathbf{l}}_w=\sum \limits_{t=1}^T{\alpha}_t{\mathbf{h}}_t. $$

##### MLP-based module

CNN is based on phrase-level attention, which learns the enhanced local phrase information of the EMR, and BiLSTM based on word-level attention learns enhanced context information of the entire EMR. It is necessary to better integrate the two pieces of information, so an MLP-based integration module is established. The MLP module consists of the left and right branches. The left branch is the enhanced local phrase representation **l**_*r*_, the right branch is the enhanced context representation **l**_*w*_, and **l**_*F*_ is the concatenation of **l**_*r*_ and **l**_*w*_ and is defined as follows:

8 $$ {\mathbf{l}}_F=\left[{\mathbf{l}}_r,{\mathbf{l}}_w\right], $$

where [.,.] indicates the concatenation operation. **l**_*F*_ goes through a one-layer MLP to obtain a combination representation, **p**_*F*_. The fully connected layer is applied to further fuse the features within **p**_*F*_ to obtain the representation of the middle side, **s**_*F*_.

#### Combination strategy

As shown in Fig. 2, the left and right sides of the framework obtain more detailed features, which we call low-level features. The middle part is based on attention, which learns high-level features. We designed a combination strategy to obtain corresponding scores from different emphases. For the concatenation of low-level local features **z**_*C*_ of the left side and high-level combination features **p**_*F*_ of the middle part, the emphasis is placed on learning the local information of an EMR,

9 $$ {\mathbf{p}}_C=\left[{\mathbf{z}}_C,\kern0.5em {\mathbf{p}}_F\right]. $$

**s**_*C*_ is obtained after **p**_*C*_ goes through the fully connected layer, and **s**_*C*_ contains local information and context information enhanced by the phrase-level and word-level attention mechanisms. Lower-level context features **z**_*B*_ of the right side and high-level features **p**_*F*_ of the middle are concatenated, and the emphasis is placed on learning the context information of an EMR,

10 $$ {\mathbf{p}}_B=\left[{\mathbf{z}}_B,\kern0.5em {\mathbf{p}}_F\right]. $$

**p**_*B*_ also goes through a fully connected layer and outputs **s**_*B*_ which contains the context information of an EMR and enhanced local information, and its dimension is the same as the number of disease labels. **f** is the final representation of an EMR, and it is a weighted sum of **s**_*C*_, **s**_*B*_, and **s**_*F*_. It is defined as follows:

11 $$ \mathbf{f}=\alpha {\mathbf{s}}_C+\beta {\mathbf{s}}_B+\gamma {\mathbf{s}}_F, $$

where *α*, *β* and *γ* are used to control the contributions of **s**_*C*_, **s**_*B*_ and **s**_*F*_, the values of *β* and *γ* are calculated based on one half of 1 − *α* , and *α* is a hyperparameter. **f** is inputted into a softmax layer to obtain **p**,

12 $$ \mathbf{p}=\mathrm{softmax}\left(\mathbf{f}\right). $$

where **p** is a prediction probability distribution of *C* disease classes (*C* = 10). **p**_*i*_ represents the probability that an EMR is related to the *i*-th disease.

In our model, the cross-entropy loss between the ground truth distribution of disease labels and the estimated probability distribution **p** is calculated as follows:

13 $$ loss=-\sum \limits_{d\in T}\sum \limits_{c=1}^C{\mathbf{g}}_c(d)\log \left({\mathbf{p}}_c(d)\right), $$

where **g** ∈ *R*^*C*^ is a vector that contains the true classification labels. *T* represents the training sample set, and *C* is the number of diseases.

## Results

### Evaluation metrics of the model

In general, we use accuracy when evaluating the performance of the classifier. Accuracy is defined as the rate of the number of samples correctly classified by the classifier among the total number of samples for a given test dataset.

14 $$ Accuracy\kern0.5em =\frac{TP+ TN}{TP+ FP+ TN+ FN} $$

where true positive (TP): in the test set, the classifier correctly classifies the positive samples into positive classes, true negative (TN): in the test set, the classifier correctly classifies negative samples into negative classes, false positive (FP): in the test set, the classifier incorrectly classifies negative samples into positive classes, false negative (FN): in the test set, the classifier incorrectly classifies positive samples into negative classes. In terms of a specific disease, such as diabetes, an EMR with a label, diabetes, is a positive example. An EMR with any other disease labels is regarded as a negative example.

**Fig. 3:**
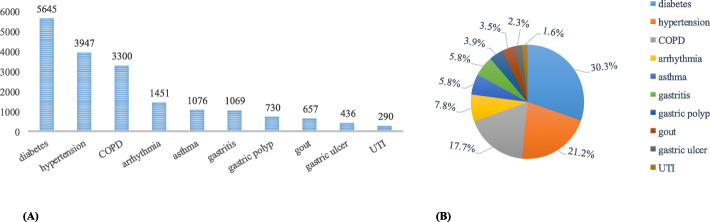
The number and proportion of each disease in the EMR set. **a** Shows the number of EMRs related to each disease. **b** Shows proportion of EMRs related to a specific disease among all the EMRs

Accuracy alone is not sufficient to measure the performance of a classifier. As shown in Fig. 3, in the dataset, *urinary tract infections* is associated with 1.6% of the EMRs, and *diabetes* is associated with 30.3% of the EMRs. There is an imbalance among the EMRs associated with one disease and those associated with another disease. Figure 3a shows the number of EMRs related to each disease, and Fig. 3b is the proportion of EMRs related to a specific disease among all the EMRs. For such an imbalance problem, the macro-average is also used to evaluate the performance of the model.

The macro-average calculates three values, *Precision*_*macro*, *i*_, *Recall*_*macro*, *i*_ and *f*_*macro,i*_ for each disease, and averages *f*_*macro,i*_ values of all the diseases. *Precision*_*macro*, *i*_ is the rate of the correctly identified positive samples (EMRs) of the *i*-th disease (called *d*_*i*_) among the samples that are retrieved. It is calculated as follows:

15 $$ {Precision}_{macro,i}=\frac{TP_{macro}}{TP_{macro}+{FP}_{macro}}, $$

where *TP*_*macro*_ is the number of successfully identified positive samples about *d*_*i*_, and *FP*_*macro*_ is the number of samples that are misidentified as *d*_*i*_. *Recall*_*macro*, *i*_ is the proportion of the d_i_-related positive samples among all samples. It is defined as follows:

16 $$ {Recall}_{macro,i}=\frac{TP_{macro}}{TP_{macro}+{FN}_{macro}}, $$

where *FN*_*macro*_ is the number of misidentified *d*_*i*_ − related samples. *f*_*macro*, *i*_ is the F ‐ score value of *d*_*i*_, and it is the harmonic average of *Precision*_*macro*, *i*_ and *Recall*_*macro*, *i*_; we obtain

17 $$ {f}_{macro,i}=\frac{2\times {Precision}_{macro,i}\times {Recall}_{macro,i}}{Precision_{macro,i}+{Recall}_{macro,i}}. $$

Finally, we calculate the average of all *f*_*macro*, *i*_ (1 ≤ *i* ≤ *C*) and obtain

18 $$ \mathrm{F}\hbox{-} {\mathrm{score}}_{\mathrm{macro}}=\frac{\sum \limits_{i=1}^C{f}_{macro,i}}{C}, $$

where *C* represents the number of diseases.

### Baselines

To evaluate the performance of the proposed method, FCNBLA, we compare it with several state-of-the-art methods of disease prediction. We describe them in detail as follows.

#### SVM-TFIDF

TF-IDF is a commonly used weighting technique for information retrieval and data mining. This baseline model based on TF-IDF extracted key information and formed the representations of EMRs. SVM is used to classify and predict the disease related to a specific EMR [23].

#### CNN

In the word embedding layer, this method maps each word of an EMR into a word embedding, and all word embeddings form an embedding matrix. In the convolutional layer, the matrix is scanned with different filters to obtain different local representations. After max pooling is completed, the extracted multiple representations are concatenated end to end. Finally, the fully connected layer and softmax layer are used to obtain the probability that the EMR is associated with a disease [10].

#### RCNN

This method differs from the traditional CNN, and it first applies a bi-directional recurrent structure to capture the contextual information to the greatest extent possible when learning word representations. Second, the max pooling layer is used to form a more effective semantic representation. The representation is utilized to predict the disease related to an EMR [45].

#### BiLSTM

To use the context information between words in the sentence, we also established a baseline method based on BiLSTM. Each word in an EMR is mapped into a word embedding through the word embedding layer, the word sequence is inputted into BiLSTM to obtain the hidden representation of any word, and the association probability is obtained. We compared our method, FCNBLA, with the baseline.

### Parameter setting

Word embeddings that are inputted into the convolutional layer are the same as those that are fed into the BiLSTM layer. Our word embedding is initialized with uniform samples from $\left[-\sqrt{3/d},\kern0.5em +\sqrt{3/d}\right]$, where we set *d* = 300. In the convolutional module, we use three different filter heights *k* ∈ [2, 3, 4]. The hidden layer dimension of the LSTM is 200, and the BiLSTM eventually outputs a 400-dimensional sentence representation. The Adam optimization algorithm is used to update the parameters, and the learning rate is set to 0.001. We apply a dropout strategy to the embedding layers of CNN and BiLSTM; the dropout rate is 0.2, and the batch size is 16. The value of *α* is 0.3, early stopping is adopted, and its value is set to 20 and in training, we used 100 epochs. For the support vector machine (SVM) method, the term frequency-inverse document frequency (TF-IDF) is used to extract features from EMRs. The document frequency is set to 5, which means that terms that appear in fewer than 5 documents are ignored. The value of n-gram ranges between 1 and 3. For the CNN, each word is also mapped to a 300-dimensional dense vector, which is randomly initialized. The heights of the filters are 4, 5, and 6, and each height has 128 filters. To ensure the fairness of the experiment, we also use randomly initialized word embeddings for RCNN, and the hidden layer size is 100. For the competing model BiLSTM, the hidden layer dimension of LSTM is set to 150. The learning rate of all competing models is 0.001, and their epochs are 100. Our implementation uses PyTorch and Python 3.6 to train and optimize the neural networks, and we use GPU cards (Nvidia GeForce GTX 1080) to speed up the model training process.

### Result comparisons with other methods

As shown in Table 2, we can see that our method achieves the best effect on each evaluation method. On the test set, our method achieves 92.78% accuracy. FCNBLA performs best in terms of macro-average results. It achieves the highest *precision*_*macro*_  (92.31%), *Recall*_*macro*_  (90.46) and F ‐ score_macro_ (91.29%), and its F ‐ score_macro_ is 3.27, 2.37, 0.75 and 1.19% higher than SVM-TFIDF, CNN, RCNN and BiLSTM, respectively. The performance of SVM-TFIDF is worse than that of the other methods. A main reason is that SVM-TFIDF is a shallow model, which fails to deeply learn the complex feature representations of EMRs. CNN only focuses on local information contained by several words, which makes its F ‐ score_macro_ lower than BiLSTM. RCNN is the second-best performing method. This means that both context information and local information are very important for the association between EMRs and diseases. BiLSTM is slightly lower than RCNN because it only learns the context information formed by word sequences.

**Table 2:** Prediction result of FCNBLA and its baselines on the test set

Methods	*Precision*_*macro*_(%)	*Recall*_*macro*_(%)	F ‐ score_macro_(%)	*Accuracy* (%)
FCNBLA	**92.31**	**90.46**	**91.29**	**92.78**
SVM-TFIDF	88.37	87.73	88.02	90.76
CNN	89.94	89.40	89.64	91.97
RCNN	91.21	89.97	90.54	92.51
BiLSTM	90.91	89.45	90.10	92.13

As shown in Table 3, 10 diseases are listed on the left side in descending order of data volume (the specific quantity of data for each disease is shown in Table 1). We list the macro-average *F-score* value corresponding to each disease. FCNBLA achieved the highest *F-score* value in 8 of the 10 diseases. In terms of the diseases with large quantities of data, FCNBLA shows a slight improvement in performance compared to other baseline models, such as diabetes, COPD, and arrhythmia, which improve slightly, by approximately 0.1 to 0.5%. However, there are significant improvements in the diseases with fewer data, such as UTI, gastritis, and gastric polyps, which improve by 2.17, 2.19, and 1.08%, respectively, compared to the best baseline model.

**Table 3:** Prediction result for each disease of FCNBLA and its baselines on the test set

*F-score* (%)	FCNBLA	SVM_TFIDF	CNN	RCNN	BiLSTM
Diseases					
diabetes	**96.29**	95.27	96.08	96.22	95.93
hypertension	89.24	87.46	88.72	89.37	89.30
COPD	**96.72**	96.57	96.64	96.66	96.49
arrhythmia	**87.68**	84.88	87.35	87.23	86.35
asthma	93.59	92.06	92.81	94.56	93.43
gastritis	**83.25**	74.82	81.36	79.90	79.12
gastric polyps	**89.90**	84.77	88.74	88.37	88.82
gout	**91.89**	90.35	90.24	91.76	90.15
gastric ulcers	**90.48**	84.75	89.39	89.94	89.66
UTI	**93.91**	89.29	91.08	91.38	91.74

RCNN performs the best for the disease *hypertension*, and its’ F ‐ score_macro_ is only 0.13% higher than our model, it indicates RCNN is just slightly better than our model for the disease. For the disease *asthma*, the F ‐ score_macro_ of RCNN is 0.97% higher than our model. We calculated the proportion of the number of EMRs in the corresponding word number range for each disease among the total number of EMRs for that disease and listed it in the supplementary table ST1. We found that EMRs with more than 500 words accounts for 72.77% of the total EMRs for asthma, while among other diseases, the highest proportion of EMRs with more than 500 words is 31.03%. It shows that RCNN performs better than our method and the other compared methods for the EMRs with more than 500 words. The primary reason is that RCNN uses the context information of left and right sides of a word to enhance the representation of the word, and the less information is lost during the process of learning extremely long text.

## Discussion

### Effect of attention at the phrase level and the word level

To validate the effect of phrase-level attention and that of word-level attention, we also implemented an instance of FCNBLA, which only has an attention mechanism at the phrase level (FCNA). Similarly, an instance that has only attention at the word level (FBLA) and another instance that has no attention (FNOA) are constructed. As shown in Table 4, F ‐ score_macro_ values of FCNA (89.49%) and FBLA (89.72%) are 0.59 and 0.82% higher than FNOA, respectively. Compared with FNOA, their accuracy is increased by 0.19 and 0.67%, respectively. This result indicates that establishing both the attention phase level and the word level is helpful for improving the performance of disease prediction.

**Table 4:** Prediction results of FCNBLA and its three instances FNOA, FCNA and FBLA

Methods	*Precision*_*macro*_(%)	*Recall*_*macro*_(%)	F ‐ score_macro_(%)	*Accuracy* (%)
FCNBLA	92.31	90.46	91.29	92.78
FNOA	89.78	88.20	88.90	91.49
FCNA	88.80	90.37	89.49	91.68
FBLA	90.98	88.66	89.72	92.16

Phase-level attention is exploited to enhance the local information, and word-level attention is used to capture the context information. For the results of F ‐ score_macro_ and accuracy, FBLA is slightly higher than FCNA. This indicates that the context information is more effective than the local information in enhancing EMR representations. A possible reason is that the phrase information learned can reflect local features of EMRs, but a comprehensive understanding of the context relationships of all the words can extract more information from a given EMR. Compared with FCNA and FBLA, the F ‐ score_macro_ of FCNBLA is increased by 1.80 and 1.57%, and its accuracy is increased by 1.10 and 0.62%, respectively. This confirms that it is necessary to introduce these two attentions.

### Effect of the combination features of the middle module

To verify the effect of using the combination features learned by the CNN module and BiLSTM module, we remove the entire middle module based on MLP. The new instance is referred to as CNBL. CNBL consists of the left side and the right side. The local representation is learned by the left side, and the context representation is learned by the right side. Similar to the integration strategy of the three sides, the final prediction is obtained by integrating these two sides. As shown in Table 5, FCNBLA is 1.77 and 0.92% higher than CNBL on the F ‐ score_macro_ and accuracy, which confirms the importance of the middle module for deeply combining local information and context information in terms of performance improvement.

**Table 5:** Prediction results of FCNBLA and its instance CNBL

Methods	*Precision*_*macro*_(%)	*Recall*_*macro*_(%)	F ‐ score_macro_(%)	*Accuracy* (%)
FCNBLA	92.31	90.46	91.29	92.78
CNBL	89.76	89.72	89.52	91.86

### Effect of our pairwise combination strategy

To verify the effect of our pairwise combination strategy, we implement an instance of FCNBLA, which is called TCNBLA. TCNBLA consists of the left side, the right side and the middle. It concatenates all local information **z**_*C*_ obtained by the left side, the context information **z**_*B*_ obtained by the right side, and the enhanced combination information **p**_*F*_ obtained by the middle module. Finally, the concatenation of the three pieces of information is fed into the fully connected and softmax layers to obtain a prediction result. As shown in Table 6, FCNBLA is 1.18 and 0.38% higher than TCNBLA on the F ‐ score_macro_ and accuracy, which proves that the semantic representation of an EMR learned from different emphases has an important role in improving performance.

**Table 6:** Prediction results of FCNBLA and its instance TCNBLA

Methods	*Precision*_*macro*_(%)	*Recall*_*macro*_(%)	F ‐ score_macro_(%)	*Accuracy* (%)
FCNBLA	92.31	90.46	91.29	92.78
TCNBLA	90.70	89.64	90.11	92.40

## Conclusions

A new method based on CNN and BiLSTM, FCNBLA, is developed for predicting the disease related to a given EMR. We establish attention mechanisms at the phrase and word levels to discriminate the different contributions of each phrase and word. This new framework is composed of three parts and is constructed for learning the local representation, context representation and combination representation enhanced by the attention mechanisms. In our experiments, the results show that FCNBLA is superior to other methods not only for macro-average but also for accuracy. Experimental results also confirm that phrase-level and word-level attention mechanisms and combination representation can enhance the inference of the disease related to a given EMR. FCNBLA may give scores for the diseases related to an EMR, and these scores are used to rank candidate diseases. FCNBLA can serve as a prediction tool to assist doctors in diagnosing diseases in patients.

## Data Availability

The datasets analyzed during the current study are downloaded from the website https://github.com/YangzlTHU/C-EMRs. Our code is available for the readers according to their reasonable request.

## Abbreviations

CNN: Convolutional neural network
BiLSTM: Bidirectional long short-term memory
EMR: Electronic medical record
RCNN: recurrent convolutional neural network
COPD: Chronic obstructive pulmonary disease
UTI: Urinary tract infections
MLP: Multilayer perceptron
SVM: Support vector machine
TF-IDF: Term frequency-inverse document frequency
TP: True positive
FP: False positive
FN: False negative
